# Vitamin D Effects on Selected Anti-Inflammatory and Pro-Inflammatory Markers of Obesity-Related Chronic Inflammation

**DOI:** 10.3389/fendo.2022.920340

**Published:** 2022-06-13

**Authors:** Maria Krajewska, Ewelina Witkowska-Sędek, Małgorzata Rumińska, Anna Stelmaszczyk-Emmel, Maria Sobol, Anna Majcher, Beata Pyrżak

**Affiliations:** ^1^Department of Paediatrics and Endocrinology, Medical University of Warsaw, Warsaw, Poland; ^2^Department of Laboratory Diagnostics and Clinical Immunology of Developmental Age, Warsaw, Medical University of Warsaw, Warsaw, Poland; ^3^Department of Biophysics, Physiology and Pathophysiology, Medical University of Warsaw, Warsaw, Poland

**Keywords:** obesity, children, interleukin-10, interleukin-17, C-reactive protein, blood leukocyte profile, platelets, vitamin D

## Abstract

**Background:**

Obesity is related to changes in adipokine secretion, activity of adipose tissue macrophages, helper T cells, and regulatory T cells. It has been confirmed that vitamin D has potent anti-inflammatory properties. It contributes to reduction in pro-inflammatory mediators and an increase in anti-inflammatory cytokines. There is also evidence that vitamin D could decrease C-reactive protein (CRP) and affect selected haematological indices.

**Aim of the Study:**

We aimed to evaluate the effect of vitamin D on interleukin (IL)-10, IL-17, CRP, blood leukocyte profile, and platelet (PLT) count in overweight and obese children before and after six months of vitamin D supplementation.

**Material and Methods:**

The study group consisted of 67 overweight and obese children aged 9.08-17.5 years. The control group included 31 normal weight peers age- and sex-matched. None of the studied children had received vitamin D supplementation before the study. Data were analyzed at baseline and after vitamin D supplementation.

**Results:**

The study group had lower baseline 25(OH)D (p<0.001) and higher white blood cell (WBC) (p=0.014), granulocyte (p=0.015), monocyte (p=0.009) and CRP (p=0.002) compared to the control group. In the study group, vitamin D levels were related negatively to nutritional status. Leukocyte profile parameters, PLT, CRP, IL-10 or IL-17 were not related to baseline 25(OH)D. Baseline IL-17 levels correlated with monocytes (R= 0.36, p=0.003) independently on 25(OH)D deficit. In children with vitamin D <15ng/ml, the baseline 25(OH)D was related to CRP (R=-0.42, p=0.017). After six months of vitamin D supplementation, we noticed a decrease in CRP levels (p=0.0003). Serum 25(OH)D correlated with IL-10 in that period (R=0.27, p=0.028). Moreover, we noticed that IL-10 correlated with monocyte (R=-0.28, p=0.023). We did not find any significant associations between 25(OH)D and leukocyte profile parameters, PLT, or IL-17. The multivariable stepwise regression analysis identified IL-10 as the parameter positively associated with 25(OH)D.

**Conclusions:**

Our study confirmed beneficial effects of vitamin D supplementation in overweight and obese paediatric populations. Vitamin D intake seems to exert its anti-inflammatory effect mainly *via* decreasing the CRP level and protecting stabile values of IL-10, rather than its impact on pro-inflammatory factors such as lL-17 and leukocyte profile parameters.

## Introduction

Vitamin D deficiency is commonly observed in overweight and obese children and adolescents (1, 2). The inverse associations between 25-hydroxyvitamin D (25(OH)D) serum levels and both fat volume and body mass index (BMI) have been confirmed (3, 4). The main mechanisms involved in the obesity-related hypovitaminosis D include decreased bioavailability of vitamin D due to its fat solubility and sequestration in abdominal fat, reduced intestinal absorption, impaired metabolism, decreased liver 25(OH)D synthesis as a result of hepatic steatosis, and the influence of leptin and interleukin-6 (IL-6) on hepatic vitamin D receptors (VDRs) (2, 3, 5–10). There is also some evidence that inflammation could reduce 25(OH)D levels *via* oxidative stress resulting in the oxidative 25(OH)D catabolism (11, 12). Sedentary lifestyle and lower outdoor physical activity, leading to insufficient sun exposure as well as inappropriate vitamin D dietary intake, also predispose obese individuals to vitamin D deficiency (8, 13). Taking into account the high prevalence of overweight and obesity in people of all age groups, including children and young adults, the role of vitamin D in the pathogenesis of obesity and prevention of obesity-related metabolic disorders is extensively investigated (2, 11, 14–17). Adipose tissue cannot be considered only as an energy reservoir that consists of adipocytes and their precursors. It also contains mesenchymal progenitor/stem cells, endothelial cells, pericytes, T cells, and M2 macrophages known as stromal vascular fraction, which play an important role in the integration of endocrine, metabolic, and inflammatory signals (2, 18). Excess body fat mass is closely related to significant changes in adipokine secretion, accumulation, and activity of adipose tissue macrophages, helper T (Th) cells, and regulatory T (Treg) cells (2, 19–24). Several studies have shown an increase in pro-inflammatory factors [IL-6, IL-8, IL-1β, IL-17, leptin, tumor necrosis factor alpha (TNF-α)] and reduction in adiponectin and anti-inflammatory interleukins (IL-4, IL-10, IL-13) in obese individuals (2, 25, 26). These mediators are involved in mutual interactions between the immune and metabolic systems, contributing to the development of insulin resistance, hyperglycaemia, atherogenic dyslipidaemia, and hypertension which highly increase the risk of atherosclerotic cardiovascular disease and diabetes mellitus type 2, even in the paediatric and young adult populations (25, 27, 28). Moreover, pro-inflammatory cytokines could affect systemic inflammation by enhancing liver production of acute phase markers including fibrinogen and C-reactive protein (CRP) and by activating granulocyte and monocyte progenitor cells (29–32). Higher blood leukocyte, lymphocyte, granulocyte, eosinophil, and monocyte count is well documented in obese individuals (33–36).

Current studies show that vitamin D exerts multiple non-calcaemic effects. Vitamin D receptors have been discovered in many cells and types of tissues, including human subcutaneous adipose tissue, visceral adipose tissue, pancreatic beta-cells, and T cells (2, 37–42). Moreover, the presence of VDRs has been also confirmed in the brain in arcuate and paraventricular nuclei of the hypothalamus, which are responsible for regulation of body weight (41, 42). Lumeng et al. (21) indicate that hypothalamic inflammation impacts metabolism, mainly by reducing the release of insulin from beta cells, impairing insulin peripheral action and also by aggravating hypertension. The main mechanisms of vitamin D action in obesity include the influence on adipose tissue inflammatory process *via* effects on adipokine secretion and on the immune system cells by regulating their proliferation and metabolism leading to inhibition of T cell proliferation and induction of Treg differentiation (17, 38, 43–46). It has been confirmed that the active form of vitamin D (1,25-dihydroxyvitamin D) has potent anti-inflammatory properties resulting in a switch from Th1/Th17 response, which is more inflammatory to Th2/Treg response, which has less inflammatory potential (11, 47–49). This results in decreased secretion of pro-inflammatory mediators such as interferon gamma (IFN-γ), TNF-α, IL-1β, IL-6, IL-8, IL-12, IL-17 and increased production of anti-inflammatory cytokines such as IL-4 and IL-10 (11, 50–54). There is also some evidence that vitamin D could decrease serum CRP levels and the erythrocyte sedimentation rate (55–61). The effect of hypovitaminosis D, as well as the influence of vitamin D supplementation on selected haematological indices has been also investigated but available data are limited (62–65). In addition to the fact that most authors reported the link between vitamin D and red blood cell parameters (66–68), there are also some studies describing the impact of vitamin D on monocyte and platelet (PLT) count (64, 69, 70). Information about those associations in obese children and adolescents is scarce.

In the present study we aimed to evaluate the effect of vitamin D on IL-10, IL-17, CRP, blood leukocyte profile, and PLT count in a group of overweight and obese children before and after six months of vitamin D supplementation.

## Material and Methods

This prospective study was conducted in the Department of Paediatrics and Endocrinology of the Medical University of Warsaw, Poland. Design of the study was approved by the Bioethics Committee at the Medical University of Warsaw, Poland (decision number KB/257/2013). The study group consisted of 67 children (15 overweight and 52 obese) aged from 9.08 to 17.5 years with mean body mass index (BMI) 30.9 ± 4.7. The control group included 31 normal weight peers with mean BMI 18.7 ± 2.7 age- and sex-matched. None of the studied children had received vitamin D supplementation within the last 12 months before being including in the study. In both the study and the control groups, blood morphology was evaluated to exclude iron deficiency anaemia due to its possible effect on platelet count. Patients with iron deficiency features in blood morphology were not included in the study. At the time of blood collection, children in both the study and the control group were healthy, without any symptoms of infection and chronic diseases and were not taking any medication. During the study period the participants did not change their diet or the level of physical activity. Vitamin D status, levels of IL-10, IL-17, CRP, blood leukocyte profile, and PLT count were determined at baseline (in the study group and in the control group) and after six months of vitamin D supplementation (in the study group).

The aim of vitamin D supplementation was to achieve the reference serum 25(OH)D levels between 30 and 50 ng/ml after six months of intervention (71). The doses of vitamin D ranged from 2000 to 4000 units per day depending on the serum 25(OH)D levels, which were assessed every month. Systematic evaluation of serum 25(OH)D concentrations allowed us to control compliance and to modify administered vitamin D doses to achieve reference values after six months of the study.

Anthropometric parameters (height, weight, waist and hip circumference) were measured using standardized methods. Based on these measurements, BMI, waist-to-hip ratio (WHR), and waist-to-height ratio (WHtR) were calculated. The skinfold thickness (mm) was measured under the triceps brachii muscle and under the inferior scapular angle. Body fat percentage was calculated in the study group and in the control group using the Slaughter formula (72). Additionally, in the study group, the percentage of fat was calculated using a bioimpedance analysis device (Maltron Body FAT Analyzer BF-905). Height and weight were evaluated according to Polish 2010 growth references for school-aged children and adolescents (73). The degree of obesity expressed as BMI standard deviation score (SDS) was calculated using the LMS method to normalize skewness of the distribution of BMI (73, 74). Obesity was defined as BMI SDS ≥ 2, and overweight as BMI SDS ≥ 1 and < 2 (75).

Data of the study group were analyzed in the whole group and in subgroups depending on baseline vitamin D status (serum 25(OH)D < 15 ng/ml - a subgroup of overweight and obese children with “severe” baseline vitamin D deficiency and serum 25(OH)D ≥ 15 ng/ml - a subgroup of overweight and obese children with “low” baseline vitamin D deficiency).

### Biochemical Analyses

Blood samples were collected after overnight fasting and analyzed by standard methods. White blood cells (WBC) and PLT count were obtained by an automated blood cell counter (XN-1000, Sysmex, Germany). The levels of CRP (mg/dl) were measured using a fixed-point immune-rate method on the Vitros 5600 analyzer (Ortho Clinical Diagnostic, New Jersey, USA). Serum 25(OH)D levels (ng/ml) were determined by the immunoassay method using Architect Analyzer (Abbott Diagnostics, Lake Forest, USA). Serum levels of IL-10 (pg/ml) and IL-17 (pg/ml) were evaluated by ELISA (R&D Systems, Minneapolis, USA) using Asys UVM 340 analyzer.

### Statistical Analysis

Statistical analysis was performed using Statistica 13.3. Data distribution was checked using the Shapiro–Wilk test. Data were presented as means with standard deviation or the median and interquartile ranges, as appropriate. Comparisons between baseline data of the study group and the control group were made using the T-test for parametric data or using the U Mann-Whitney test for non-parametric data. Analysis of changes of the same parameter at baseline and after six months of vitamin D supplementation were provided using the T-test or the Wilcoxon test, as appropriate. Correlation analysis was performed using the Spearman correlation coefficient. In further analysis, we used multivariable stepwise regression analysis to determine which inflammatory factors (model 1: IL-10, IL-17, CRP, WBC or model 2: IL-10, IL-17, CRP, monocytes) were associated with 25(OH)D levels (as dependent variable) at baseline and after six months of vitamin D supplementation.

## Results

Baseline anthropometric and biochemical characteristics of the study group and the control group are presented in **Table 1**. The study group characterized significantly lower baseline serum 25(OH)D levels compared to the control group (median 15.9 vs 23.9 ng/ml, p < 0.001). We also found significant baseline differences in leukocyte profile parameters and CRP levels between those groups, while baseline PLT count, IL-10, and IL-17 levels did not differ significantly between the study group and the control group. Baseline WBC (p = 0.014), granulocyte (p = 0.015), monocyte (p = 0.009) count and CRP levels (p = 0.002) were significantly higher in the group of overweight and obese children and adolescents.

**Table 1:** Baseline anthropometric measurements, haematological, and biochemical parameters in the study group and in the control group.

p value	STUDY GROUP	CONTROL GROUP
	(n = 67)	(n = 31)
**Age (years)**	13.3 ± 2.11	13.8 ± 2.45
ns		
**Height SDS**	0.7 ± 1.26	-0.6 ± 1.37
< 0.001		
**Weight SDS**	2.3 ± 0.66	-0.3 ± 1.09
< 0.001		
**WC (cm)**	91.3 ± 10.17	62.3 ± 6.49
<0.001		
**HC (cm)**	106.1 ± 10.48	78.7 ± 8.86
< 0.001		
**WHR**	0.9 ± 0.06	0.8 ± 0.04
< 0.001		
**WHtR**	0.9 ± 2.68	0.4 ± 0.03
< 0.001		
**% FAT (skinfolds)**	36.9 ± 6.12	22.7 ± 6.06
< 0.001		
**BMI SDS**	2.3 ± 0.46	-0.2 ± 0.83
< 0.001		
**25(OH)D (ng/ml)**	15.9 (12.6 – 20.0)	23.9 (17.7 – 29.9)
< 0.001		
**WBC (cells × 10^3^/μl)**	6.7 (5.5 – 8.1)	5.7 (5.0 – 6.7)
0.014		
**Granulocytes (cells × 10^3^/μl)**	3.2 (2.4 – 4.5)	2.8 (1.9 – 3.7)
0.015		
**Monocytes (cells × 10^3^/μl)**	0.5 (0.5 – 0.7)	0.4 (0.4 – 0.6)
0.009		
**Lymphocytes (cells × 10^3^/μl)**	2.4 (2.1 – 2.9)	2.2 (1.9 – 2.9)
ns		
**PLT (cells × 10^3^/μl)**	270 (236 – 302)	260 (231 – 284)
ns		
**CRP (mg/dl)**	0.5 (0.5 – 0.7)	0.5 (0.5 – 0.5)
0.003		
**IL-10 (pg/ml)**	1.25 (0.03 – 1.74)	0.65 (0.19 – 1.15)
ns		
**IL-17 (pg/ml)**	0.05 (0.0 – 0.13)	0.05 (0.0 – 0.12)
ns		

Data are presented as means ± standard deviations score or as median with interquartile range, as appropriate. SDS, standard deviation score; WC, waist circumference; HC, hip circumference; WHR, waist-to-hip ratio; WHtR, waist-to-height ratio; % FAT (skinfolds), percentage of body fat estimated from skinfolds; BMI, body mass index; 25(OH)D, 25-hydroxyvitamin D; WBC, white blood cells; PLT, platelets; CRP, C-reactive protein; IL-10, interleukin-10; IL-17, interleukin-17; ns, not significant.

Severe baseline vitamin D deficiency was found in 54% (36 participants) of the study group. Taking into account baseline vitamin D status of the study group we did not find any significant differences in anthropometric and biochemical parameters between subgroups with lower and severe vitamin D deficiency.

### Baseline Associations Between Vitamin D Status, Nutritional Status, and Biochemical Parameters in the Study Group and in the Control Group

**Figure 1:**
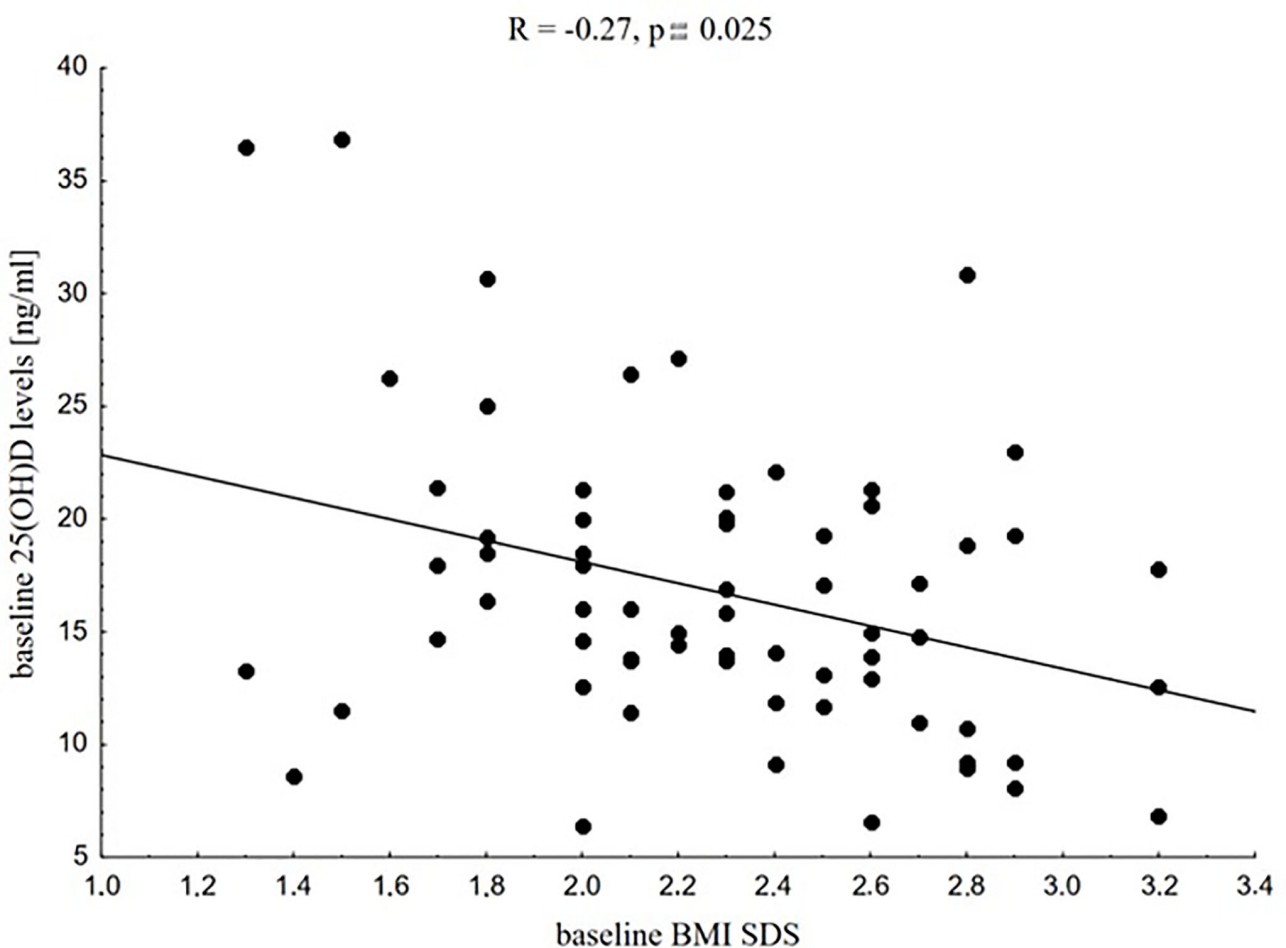
Correlation between baseline 25(OH)D levels and baseline BMI SDS in the whole study group.

At baseline, as expected, we found significant associations between vitamin D status and nutritional status, especially in children with excess body fat. In the study group, vitamin D levels were related negatively to body mass SDS (R = -0.27, p = 0.029), BMI SDS (R = -0.27, p = 0.025, **Figure 1**) and hip circumference (R = -0.26, p = 0.039). In the control group those associations were not seen, apart from the negative association between vitamin D status and hip circumference (R = -0.52, p = 0.014). Leukocyte profile parameters, PLT count, CRP, IL-10, and IL-17 levels were not related to baseline vitamin D status in the study group nor in the control group.

**Figure 2:**
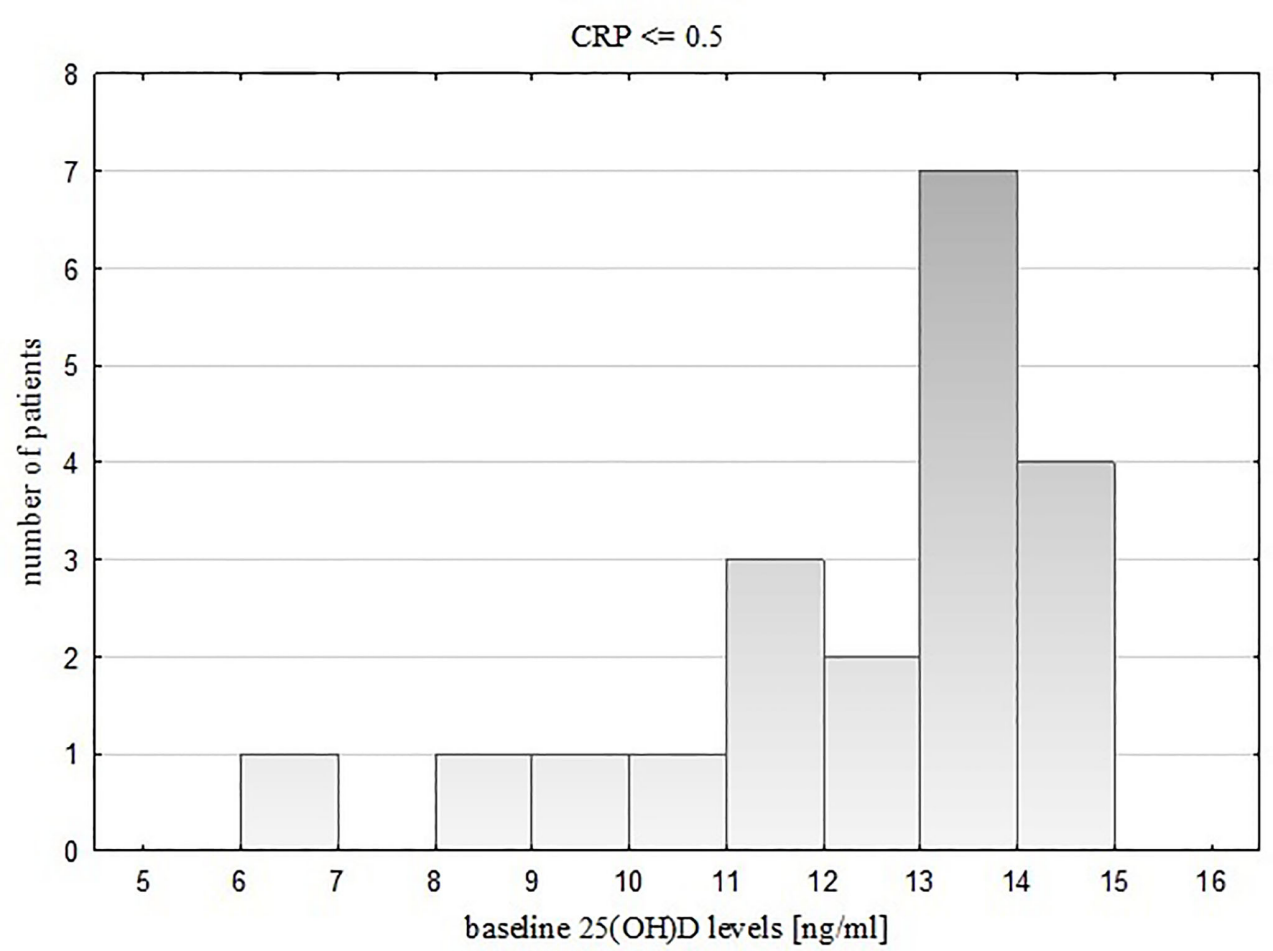
Distribution of vitamin D values in the subgroup with severe 25(OH)D deficiency and CRP <= 0.5.

**Figure 3:**
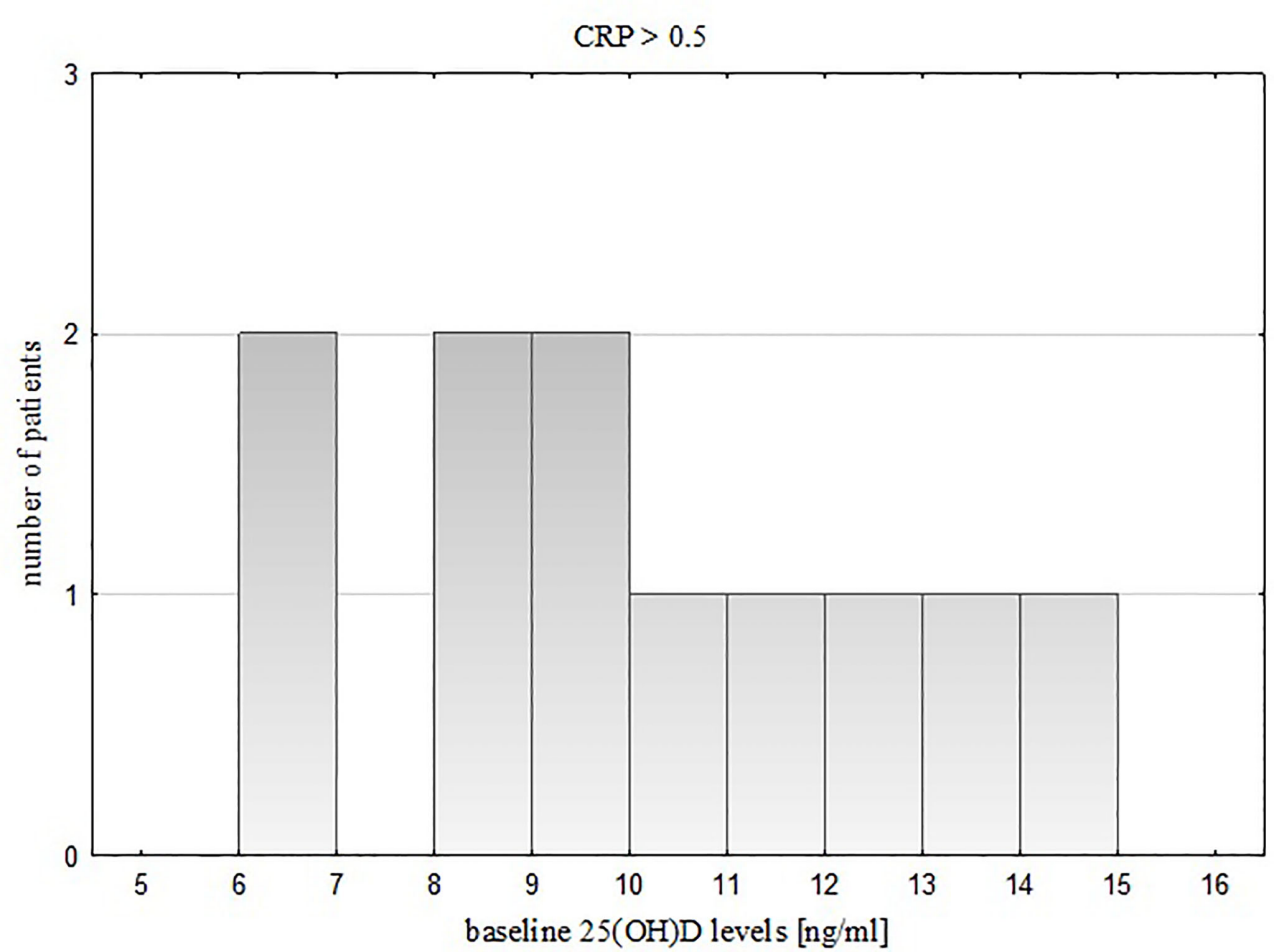
Distribution of vitamin D values in the subgroup with severe 25(OH)D deficiency and CRP > 0.5.

Taking into account baseline vitamin D status of the study group (serum 25(OH)D level ≥ 15 ng/ml or < 15 ng/ml), we noticed that in children with severe vitamin D deficiency, 25(OH)D levels were related negatively to CRP levels (R= -0.42, p = 0.017). The distribution of serum 25(OH)D values in this subgroup is presented on two histograms (**Figure 2**. for participants with CRP ≤ 0.5 mg/dl, **Figure 3** for participants with CRP > 0.5 mg/dl).

In further analysis, we also found that in this subgroup nutritional status parameters such as BMI SDS (R = 0.61, p = 0.0003), waist circumference (R = 0.44, p = 0.014), WHR (R = 0.49, p = 0.005), %FAT BIA (R = 0.43, p = 0.017), and WHtR (R = 0.70, p = 0.00003) were significantly positively related to CRP levels.

**Figure 4:**
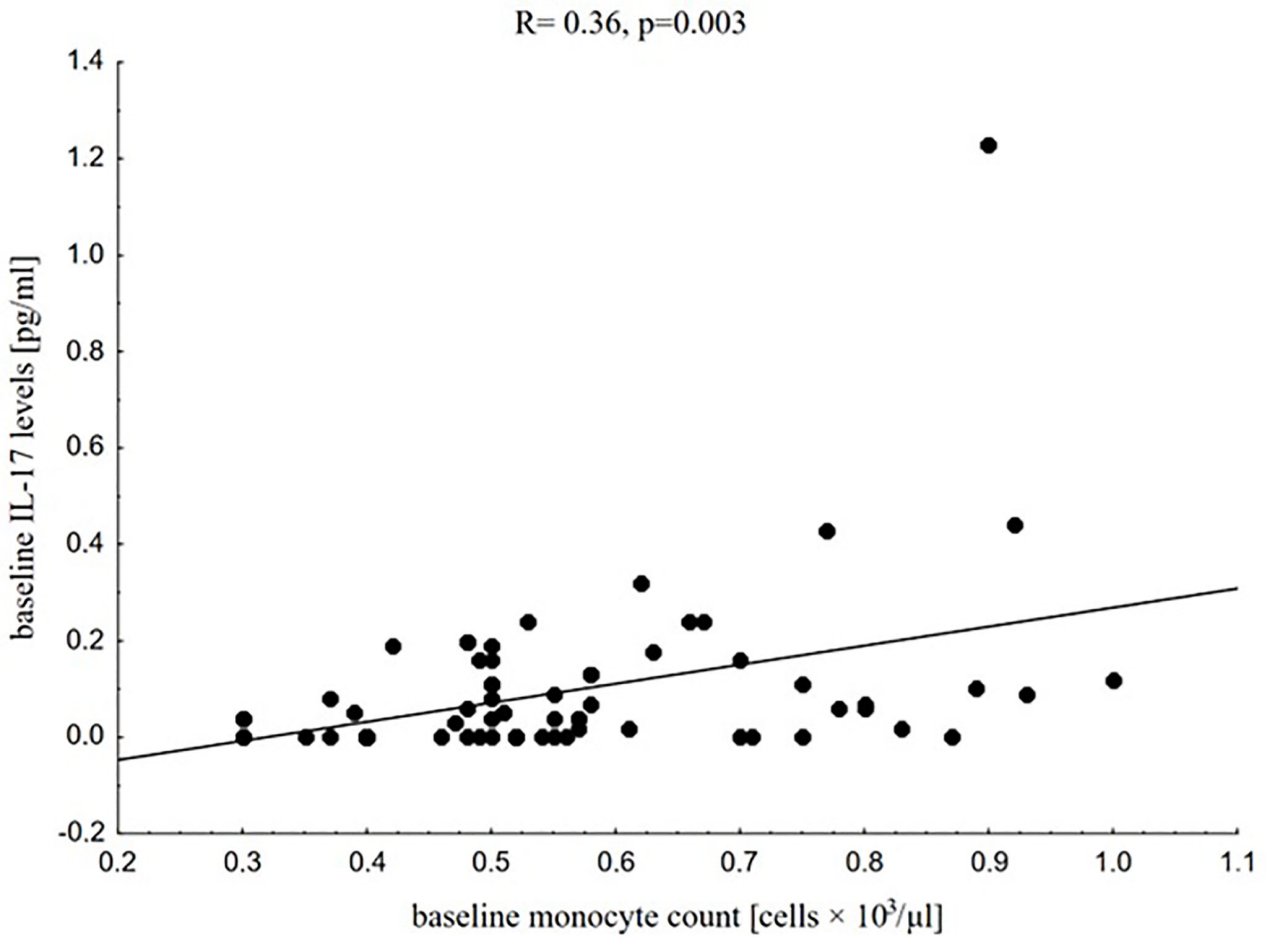
Correlation between baseline IL-17 levels and baseline monocyte count in the whole study group.

These relationships were not seen in the subgroup with low baseline 25(OH)D deficiency and in the control group. In a subgroup with low 25(OH)D deficiency we found only positive associations between hip circumference and WBC count (R = 0.47, p = 0.006) and between hip circumference and granulocyte count (R = 0.55, p = 0.001). We also observed that baseline IL-17 levels correlated positively with baseline monocytes (R = 0.36, p = 0.003, **Figure 4**) in the whole study group independently on baseline 25(OH)D deficiency (R = 0.36, p = 0.032; R = 0.37, p = 0.038, respectively in subgroups with low and severe baseline vitamin D deficiency).

### Effects of Vitamin D Supplementation on Leukocyte Profile Parameters, Platelet Count, CRP, IL-10, and IL-17 Levels in the Study Group

The characteristics of the study group at baseline and after six months of vitamin D supplementation are shown in **Table 2**. After six months of vitamin D supplementation its value increased by an average of 11.3 ± 8.2 ng/ml. Simultaneously, we noticed a significant decrease in CRP levels (p = 0.0003) without any changes in leukocyte profile parameters, PLT count, or IL-10, and IL-17 levels. Serum 25(OH)D levels correlated significantly with IL-10 levels in that period (R = 0.27, p = 0.028). We did not find any significant association between 25(OH)D levels and leukocyte profile parameters, PLT count, or IL-17 levels.

**Table 2:** Comparison between anthropometric measurements, haematological, and biochemical parameters in the study group at baseline and after six months of vitamin D supplementation.

p value	BASELINE	SIX MONTHS
**Height SDS**	0.7 ± 1.26	0.6 ± 1.25
ns		
**Weight SDS**	2.3 ± 0.66	2.2 ± 0.75
0.047		
**WC (cm)**	91.3 ± 10.17	90.6 ± 11.06
ns		
**HC (cm)**	106.1 ± 10.48	106.4 ± 11.71
ns		
**WHR**	0.9 ± 0.06	0.9 ± 0.05
ns		
**WHtR**	0.9 ± 2.68	0.5 ± 0.06
< 0.001		
**% FAT (skinfolds)**	36.9 ± 6.12	34.2 ± 6.34
< 0.001		
**% FAT BIA**	40.6 ± 7.59	38.3 ± 8.93
ns		
**BMI SDS**	2.3 ± 0.46	2.2 ± 0.55
ns		
**25(OH)D (ng/ml)**	15.9 (12.6 – 20.0)	27.1 (22.9 – 32.6)
< 0.001		
**WBC (cells × 10^3^/μl)**	6.7 (5.5 – 8.1)	6.2 (5.6 – 7.8)
ns		
**Granulocytes (cells × 10^3^/μl)**	3.2 (2.4 – 4.5)	3.3 (2.4 – 4.0)
ns		
**Monocytes (cells × 10^3^/μl)**	0.5 (0.5 – 0.7)	0.5 (0.4 – 0.6)
ns		
**Lymphocytes (cells × 10^3^/μl)**	2.4 (2.1 – 2.9)	2.3 (2.0 – 2.8)
ns		
**PLT (cells × 10^3^/μl)**	270 (236 – 302)	261 (232 – 311)
ns		
**CRP (mg/dl)**	0.5 (0.5 – 0.7)	0.5 (0.5 – 0.6)
0.001		
**IL-10 (pg/ml)**	1.25 (0.03 – 1.74)	0.78 (0.34 – 1.58)
ns		
**IL-17 (pg/ml)**	0.05 (0.0 – 0.13)	0.04 (0.01 – 0.10)
ns		

Data are presented as means ± standard deviations score or as median with interquartile range, as appropriate. SDS, standard deviation score; WC, waist circumference; HC, hip circumference; WHR, waist-to-hip ratio; WHtR, waist-to-height ratio; %FAT (skinfolds), percentage of body fat estimated from skinfolds; %FAT BIA, percentage of body fat estimated using bioelectrical impedance analysis method; BMI, body mass index; 25(OH)D, 25-hydroxyvitamin D; WBC, white blood cells; PLT, platelets; CRP, C-reactive protein; IL-10, interleukin-10; IL-17, interleukin-17; ns, not significant.

We also noticed that after six months of intervention, IL-10 levels correlated significantly negatively with monocyte count in that period (R = -0.28, p = 0.023).

In further investigation we used multivariable stepwise regression analysis to determine which inflammatory factors are associated with serum 25(OH)D levels (as dependent variable) at baseline and after six months of vitamin D supplementation.

We did not find any significant relationships in multivariable stepwise regression models including baseline 25(OH)D levels and chosen baseline inflammatory parameters as independent variables (model 1: IL-10, IL-17, CRP, WBC; model 2: IL-10, IL-17, CRP, monocytes).

After six months of vitamin D supplementation, the multivariable stepwise regression analysis, that included 25(OH)D levels (as dependent variable) and IL-10, IL-17, CRP, WBC (model 1) or IL-10, IL-17, CRP, monocytes (model 2) as independent variables, identified IL-10 as the parameter significantly positively associated with 25(OH)D levels. Both models were significant with cumulative R^2^ = 0.12, p = 0.004 and the received correlations coefficients were respectively equal β = 0.344 ± 0.116.

## Discussion

In our study we focused on the relationships between vitamin D status, both baseline and after six months of vitamin D supplementation and selected anti-inflammatory and pro-inflammatory markers. We found significant associations between serum 25(OH)D levels and levels of CRP and IL-10, while leukocyte profile parameters and PLT count, as well as IL-17 levels, seemed not to be vitamin D-dependent. As expected, we confirmed significant negative relationships between 25(OH)D levels and nutritional status parameters. Overweight and obese children from the study group had significantly lower serum baseline 25(OH)D levels compared to age- and sex-matched healthy peers, despite the lack of vitamin D supplementation in both groups before the initiation of the study. Obesity-dependent hypovitaminosis D has been previously confirmed in many studies in paediatric and adult population (3, 19, 76). Low-grade chronic inflammation characteristics for obese individuals seem to be involved with vitamin D deficiency in this group (11, 12). Analyzing baseline values of selected inflammatory markers in both groups, we noticed that WBC, granulocyte, and monocyte count, as well as CRP levels, were significantly higher in the study group than in the control group. Similar results were presented in our previously published study which considered almost 100 overweight and obese children. Our previous research indicated that WBC and granulocyte count were related to BMI SDS, while monocyte count was related to waist circumference, which could suggest that visceral adipose tissue has much greater pro-inflammatory potential than subcutaneous tissue, as a source of pro-inflammatory adipokines and cytokines (36).

The main findings of our present study regarded the interactions between vitamin D and serum levels of IL-10 and CRP and could support hypothesis of anti-inflammatory vitamin D properties. Despite this, we did not find any baseline associations between vitamin D status and leukocyte profile parameters, PLT count, CRP, IL-10, and IL-17 levels in the whole study group, and we noticed that in participants with severe baseline vitamin D deficiency (serum 25(OH)D values below 15 ng/ml) 25(OH)D levels were inversely related to CRP values. Moreover, in this subgroup, CRP levels correlated positively with nutritional status parameters. Similar associations were reported by Rodriguez et al. (77) who examined more than one hundred Spanish overweight and obese children from 9 to 12 years of age and reported that low serum 25(OH)D levels were significantly associated with increased high sensitive CRP (hs-CRP). In the study by Bellia et al. (61), based on a cohort of 147 severely obese patients with mean BMI 43.6 ± 4.3 kg/m^2^ who were prepared to bariatric surgery, a multivariate regression analysis showed that serum 25(OH)D was inversely related to hs-CRP levels, even after accounting for age, gender, season of recruitment, BMI, total body fat, and truncal fat mass. The cross-sectional analysis by de Oliveira et al. (78) based on data of 5,870 adult participants from the English Longitudinal Study of Ageing (ELSA) showed an inverse relationship not only between serum levels of 25(OH)D and CRP values but also between 25(OH)D and WBC count. In our analysis WBC count did not depend on vitamin D status but the protocols of our study and the study by de Oliveira et al. (78) were not exactly consistent. The main differences between our study and ELSA included the age of studied groups (children and adolescents in our study vs adult patients 50 years of age and over), the duration of obesity and associated inflammation (possibly much longer in de Oliveira group), differences in the number of participants (67 in our study vs almost 6,000 in ELSA). The study by Palaniswamy et al. (19) based on a cohort of 3,586 individuals with mean BMI 24.8 kg/m2 and mean 25(OH)D levels 50.3 nmol/L also confirmed negative associations between 25(OH)D and hs-CRP levels, which were simultaneously positively associated with BMI. Those findings are strictly in-line with our observations. Conversely, Palaniswamy et al. (19) concluded that their large observational and Mendelian randomization study, which analyzed the associations between 25(OH)D, BMI, and 16 inflammatory biomarkers (including IL-17, IL-1α, IL-1β, IL-4, IL-6, IL-8, TNF-α and hs-CRP), considered together with data from review of randomized controlled trials, did not confirm the beneficial role of vitamin D supplementation in obesity-related inflammation. Similar observations were previously reported by Shea et al. (79) based on data from the Framingham Offspring Study. The lack of association between vitamin D and IL-17 levels is in-line with our findings. Interestingly, we observed baseline positive correlation between IL-17 levels and monocyte count, which was independent from the severity of baseline vitamin D deficiency and could confirm mutual associations between pro-inflammatory interleukins and monocytes enhancing their inflammatory potential.

In our study we also analyzed the impact of vitamin D supplementation on selected markers of inflammation. Our study revealed that six months of vitamin D supplementation led to a significant decrease in CRP levels, influenced IL-10 levels, but did not affect leukocyte profile, PLT count, and IL-17 levels. Interestingly, those effects were observed despite the lack of significant changes in body fat mass and BMI in our study group. Data from animal and human studies regarding the impact of vitamin D supplementation on obesity-related inflammation are contradictory. The rat study by Gomma et al. (80) showed that vitamin D administration in rats that received high fat diet (HFD) led to significant decrease in body weight gain, decrease in serum CRP levels, and significant increase in serum IL-10 levels in comparison with HFD-rats that did not receive vitamin D supplementation. Mirzavandi et al. (81) who investigated the effect of vitamin D intramuscular megadose injections (200 000 IUs at baseline and next at week 4 of intervention) reported significant decrease in CRP levels in vitamin D deficient adults with diabetes mellitus type 2. Similar results, also in patients with diabetes mellitus type 2, were shown by Mousa et al. (55), who provided a systematic review and meta-analysis including twenty trials with a total of 1,270 individuals. The authors reported that vitamin D supplemented patients had lower CRP and TNF-α levels, lower erythrocyte sedimentation rate, and higher leptin levels compared to the control groups. Conversely, a systematic review with meta-analysis by Jamka et al. (82), who assessed changes in 25(OH)D and CRP levels in 1,955 obese and overweight subjects, showed that vitamin D supplementation did not affect CRP levels. Systematic review and meta-analysis of randomized control trials (RCTs) by Mazidi et al. (83) also indicated that vitamin D supplementation had no impact on serum CRP, IL-10, and TNF-α levels but the authors recommend RCTs with longer period of follow-up time (12 months) for future studies to provide explicit results.

Based on our data we noticed that serum 25(OH)D values, achieved after six months of vitamin D supplementation, correlated significantly positively with IL-10 levels in that period. Those relationships found firstly, using the Spearman correlation, was also confirmed in a multivariable stepwise regression analysis taking 25(OH)D levels as dependent variables and IL-10, IL-17, CRP, WBC (model 1), IL-10, IL-17, CRP, and monocytes (model 2) as independent variables. In both models we identified IL-10 levels measured at six months of intervention as the parameter significantly positively associated with 25(OH)D levels. Moreover, we found that IL-10 levels were inversely related to monocyte count also evaluated at six months of vitamin D intake. After six months of vitamin D supplementation, we did not find any relationships between vitamin D status and leukocyte profile parameters, PLT count, or IL-17 levels. The mechanisms of association between vitamin D status and both interleukins and leukocyte profile parameters are not clearly explained and literature data in this field are insufficient. Hashemi et al. (84), reported that based on a group of multiple sclerosis patients, vitamin D supplementation up-regulates IL-27 and TGF-β1 levels, which in consequence, increases the secretion of anti-inflammatory IL-10 and inhibits pro-inflammatory IL-17 production. The anti-inflammatory role of IL-27 was also reported by other authors (85, 86). However, the precise mechanism of vitamin D-dependent regulation of immune cells function is much more complicated and includes activation of various signalling cascades (2, 17). Most of the presented studies concern the adult population or animals, while the number of studies in the paediatric population is very limited.

In conclusion, our study confirmed beneficial effects of vitamin D supplementation in overweight and obese paediatric population. Vitamin D intake seems to exert its anti-inflammatory effect mainly *via* decreasing of CRP level and protecting stabile values of IL-10, rather than its impact on pro-inflammatory factors such as lL-17 and leukocyte profile parameters.

## Data Availability Statement

The original contributions presented in the study are included in the article/supplementary material. Further inquiries can be directed to the corresponding author.

## Ethics Statement

The studies involving human participants were reviewed and approved by Bioethics Committee of Medical University of Warsaw. Written informed consent to participate in this study was provided by the participants’ legal guardian/next of kin.

## Author Contributions

MK, EW-S and MR contributed to conception and design of the study. MK and MR organized the database. MK and EW-S prepared the tables. AM performed anthropometric measurements. MK and AS-E took measurements of serum IL-10 and IL-17 levels. MK, MR, EW-S and MS performed statistical analysis. MK and EW-S wrote the manuscript. BP supervised the work. All authors contributed to manuscript revision, read, and approved the submitted version.

## Conflict of Interest

The authors declare that the research was conducted in the absence of any commercial or financial relationships that could be construed as a potential conflict of interest.

## Publisher’s Note

All claims expressed in this article are solely those of the authors and do not necessarily represent those of their affiliated organizations, or those of the publisher, the editors and the reviewers. Any product that may be evaluated in this article, or claim that may be made by its manufacturer, is not guaranteed or endorsed by the publisher.
